# Évaluation d'un protocole de traitement des envenimations ophidiennes au Bénin

**DOI:** 10.48327/mtsi.v3i4.2023.451

**Published:** 2023-11-29

**Authors:** Harold TANKPINOU ZOUMENOU, Jean-Philippe CHIPPAUX, Pierre FACHEHOUN, Giambattista PRIULI, Achille MASSOUGBODJI

**Affiliations:** 1 Institut de recherche clinique du Bénin, Abomey-Calavi, Bénin; 2 Université Paris Cité, IRD, MERIT, 4 avenue de l'Observatoire, Paris, France; 3 Hôpital Saint Jean de Dieu, Tanguiéta, Bénin

**Keywords:** Envenimation, Morsure de serpent, Antivenin, Protocole thérapeutique, Efficacité, Tolérance, Hémostase, Bénin, Afrique subsaharienne, Envenomation, Snakebite, Antivenom, Therapeutic protocol, Efficacy, Tolerance, Hemostasis, Benin, Sub-Saharan Africa

## Abstract

**Introduction:**

La dose d'antivenin recommandée par le fabricant pour le traitement des envenimations ophidiennes, soit 2 ampoules d'Inoserp^®^ Pan-Africa (IPA), un antivenin polyvalent, systématiquement en première intention, n'est généralement pas appliquée en raison du coût élevé qui est à la charge exclusive du patient. Nous avons évalué sur la base de critères hématologiques (temps de coagulation sur tube sec, ou TCTS, et saignement) un protocole thérapeutique allégé consistant à n'administrer qu'une seule ampoule d'IPA au lieu de 2 ampoules, ce qui correspond à la pratique habituelle. Le renouvellement des doses suivait les recommandations du fabricant et du ministère de la Santé.

**Matériel et méthode:**

L’étude s'est déroulée dans un hôpital de première référence à Tanguiéta, au nord du Bénin. Tous les patients présentant des œdèmes avec ou sans troubles de la coagulation biologiques et/ou cliniques, ou une envenimation cobraïque, recevaient une ampoule d'IPA en intraveineuse directe lente. Un bilan clinique identique à celui de l'inclusion était réalisé 2, 4, 6, 12 et 24 heures après pour évaluer la tolérance, l'efficacité de l'IPA, ainsi que la nécessité de réadministrer l'antivenin. L'apparition, la persistance ou l'aggravation des saignements, ou de troubles neurologiques traduisant une envenimation par élapidé, entraînait systématiquement l'injection de 2 ou 4 ampoules d'IPA, respectivement. Les signes d'intolérance étaient recherchés avant et après chaque administration d'antivenin pour estimer l'incidence des effets indésirables imputables à l'antivenin.

**Résultats:**

L’étude s'est déroulée du 31 juillet au 31 octobre 2019. Nous avons reçu 53 cas de morsures de serpents sur lesquels 43 ont été inclus. L’âge médian était de 21 [IIQ: 18-31] ans et le sex-ratio (H/F) de 1,5. La médiane du délai d'admission était de 1 [IIQ: 0-2] jour. À l'admission, 32 patients (74%) présentaient des troubles de l'hémostase marqués par des saignements et/ou un TCTS anormal. La médiane de la normalisation du temps de coagulation sur tube sec était de 24 [IIQ: 4-72] heures. Le délai médian d'arrêt des saignements était de 6 [IIQ: 4-12] heures. Deux patients (5%) sont décédés des suites de l'envenimation.

**Discussion/conclusion:**

Le protocole allégé représente une économie significative du nombre d'ampoules utilisées (1,8 ± 0,4 ampoules par patient au lieu des 2,4 ± 0,2 pour le protocole standard; p = 2,6·10^-4^). Toutefois, en comparaison avec les résultats obtenus lors de l’étude clinique, l'arrêt des saignements est retardé tout comme la normalisation du TCTS aux differents temps de la surveillance. Il n'y a pas de différence significative entre l'incidence de signes d'intolérance précoce à l'antivenin dans cette étude par rapport à la précédente (16 *%* et 11%, respectivement; p = 0,79). Malgré son efficacité et l’économie réalisée, il ne nous semble pas raisonnable de recommander le protocole allégé.

## Abstract (en)

**Introduction:**

Snakebite envenomation is a major public health issue in Sub-Saharan Africa (SSA). Antivenoms are the only etiological treatment. However, the dose recommended by the manufacturer (2 vials renewed every 2 hours in case of persistent bleeding) is usually not applied due to the high cost which is borne only by the patient. Based on clinical presentation, we evaluated the administration of a single vial on admission (instead of 2 vials), which corresponds to the therapeutic protocol used in current practice in most health centers in Benin and beyond in SSA. Renewal of antivenom followed manufacturer's recommendation.

**Material and method:**

The study took place in a first referral hospital in Tanguiéta, North Benin. All envenomed patients received a slow intravenous vial of Inoserp^®^ Pan-Africa (IPA) upon arrival. A clinical assessment identical to that of inclusion was carried out 2, 4, 6, 12 and 24 hours later to assess the tolerance and efficacy of the IPA, as well as the need to readminister the antivenom. The occurrence, persistence or worsening of clinical hemorrhages or neurological disorders, the latter reflecting envenomation by elapids, systematically led to the injection of 2 or 4 vials of IPA, respectively. Signs of intolerance were sought before and after each administration of antivenom, then 2 weeks to 1 month after treatment, in order to estimate the incidence of adverse effects attributable to the antivenom. Efficacy of IPA was assessed by cessation of bleeding and/or normalization of whole blood coagulation test (WBCT) within 24 hours of initial administration.

**Results:**

The study took place from July 31 to October 31, 2019. We received 53 cases of snakebites of which 43 were included. The median age was 21 [IQR: 18-31] years and the sex ratio (M/F) was 1.5. Farmers were the majority (48%). The median time to admission was 1 [IQR: 0-2] day. On admission, 32 patients (74%) presented hemostasis disorders marked by bleeding and/or abnormal WBCT. None of the patients showed neurological symptoms. The median time to normalization of WBCT was 24 [IQR: 4-72] hours. The median time to stop bleeding was 6 [IQR: 4-12] hours. In 3 of the 11 patients whose bleeding had stopped at H2, a recurrence occurred within the following 24 hours. In addition, 3 patients presented with late bleeding 24 hours after admission. Two patients (5%) died as a result of envenomation.

**Discussion/conclusion:**

The simplified protocol represents a significant saving in the number of vials used, i.e. 1.8±0.4 vials per patient instead of the 2.4±0.2 which would have been necessary in these same patients treated according to the standard protocol (p = 2.6·10^-4^). However, in comparison with the results obtained during the previous clinical study, the cessation of bleeding is delayed, as is the normalization of the WBCT at the different follow-up times. In addition, the number of re-administrations of antivenom after H2 is higher in the light protocol than in the standard one, respectively 13 patients out of 43 treated versus 4 patients out of 59 (p = 0.006). There was no significant difference between the incidence of signs of early antivenom intolerance in this study compared to the previous one (16% and 11%, respectively; p = 0.79). This delay can put up to a third of patients at risk of complications. Despite its effectiveness and the antivenom savings made, it does not seem reasonable to recommend it.

## Introduction

Les envenimations par morsure de serpent représentent un grave problème de santé publique négligé dans la plupart des régions tropicales, particulièrement dans les pays en développement. En Afrique subsaharienne (ASS), plus de 300 000 envenimations par morsure de serpent sont traitées chaque année dans des centres de santé [4]. Au Bénin, l'incidence annuelle des envenimations est estimée à 200 pour 100 000 habitants ruraux – soit plus de 10 000 cas par an – avec une mortalité proche de 10 pour 100 000 – environ 700 décès – et un nombre d'invalidités définitives du même ordre de grandeur [3]. Les morsures surviennent majoritairement dans le nord du pays où elles sont significativement plus graves qu'au sud [15].

Les antivenins constituent le seul traitement étiologique. L'Inoserp” Pan-Africa (IPA) fabriqué par Inosan Biopharma est le seul antivenin actuellement commercialisé à avoir obtenu une autorisation de mise sur le marché au Bénin. Il s'agit d'un antivenin polyvalent, lyophilisé couvrant les principales espèces venimeuses de la région. Il a fait l'objet de plusieurs études cliniques montrant son efficacité et sa très bonne tolérance tant au Bénin que dans d'autres pays d'ASS [7, 12].

Le protocole thérapeutique recommandé par la Société africaine de venimologie (SAV) et adopté par le ministère de la Santé du Bénin prévoit l'administration par voie veineuse d'une ampoule d'antivenin en cas d’œdème sans trouble de la coagulation, de 2 ampoules en cas de troubles hémorragiques biologiques et/ou cliniques, et de 4 ampoules lors d'une envenimation cobraïque (troubles sensorimoteurs, paralysie respiratoire). La persistance ou l'aggravation des troubles hémorragiques ou neurotoxiques 2 heures après l'administration de l'antivenin, constitue une indication au renouvellement de l'antivenin à la même dose [5].

Cependant, il est rapidement apparu que cette posologie était difficile à respecter en raison du coût élevé de l'antivenin (environ 50 € par flacon) à la charge exclusive du patient. La pratique a montré que, d'une part une seule dose était administrée en première intention quels que soient les symptômes présentés par le patient et, d'autre part le renouvellement n’était envisagé que 24 heures après son administration en cas de détérioration de l’état clinique du patient et non 2 heures après comme le prévoit le protocole standard. La présente étude avait pour objectif principal d’évaluer, selon des critères standardisés, l’évolution clinique de l'envenimation 2 heures après l'administration d'une seule ampoule d'IPA.

## Méthode d’étude

### Cadre de l’étude

L’étude s'est déroulée à l'hôpital Saint Jean de Dieu de Tanguiéta, situé dans le nord du Bénin. Il fait partie des hôpitaux de références de la région, et dessert une population estimée à 265 000 habitants. Chaque année, l'hôpital de zone de Tanguiéta reçoit environ 200 cas de morsures de serpents.

### Population étudiée

Il s'agissait d'une étude prospective, descriptive réalisée sur une période de 3 mois, du 31 juillet au 31 octobre 2019. Elle incluait tous les patients victimes d'une morsure de serpent survenue au cours des 7 jours précédents et présentant au moins un signe patent d'envenimation (œdème, saignement, anomalie du test de coagulation sur tube sec (TCTS) et/ou troubles neurologiques). Les patients ayant reçu un antivenin avant leur admission à l'hôpital n'ont pas été inclus dans l’étude.

### Collecte de données

À l'admission, la gravité de l'envenimation était mesurée à l'aide de scores cliniques (œdème, hémorragie et neurotoxicité: Tableau I) et d'un TCTS réalisé au lit du malade [1, 6]. Le seul examen biologique qui était systématique était un hémogramme à l'entrée du patient.

**Tableau I:** Score de gravité de l'envenimation par signes cliniques [d'après 5] Envenomation severity score according to clinical signs [after 5]

Grade	Œdème	Syndrome hémorragique	Syndrome neurologique
**0**	Pas d’œdème	Pas de saignement	Pas de signe décrit
**1**	Œdème localisé (ne dépasse pas le poignet ou la cheville)	Saignement local persistant plus d'une heure	Anesthésie locale, fourmillement, picotement du membre mordu, fasciculation
**2**	Œdème régional (ne dépasse pas le coude ou le genou)	Saignement muqueux, saignement des cicatrices anciennes	Troubles sensorimoteurs à la tête (audition, vue, goût, dysphagie)
**3**	Œdème atteint tout le membre mordu	Ecchymose étendue, hématomes distants, purpura, phlyctènes distantes	Troubles respiratoires
**4**	Œdème dépassant la racine du membre	Hémorragie interne	Troubles de la vigilance

### Traitement

Nous avons utilisé des ampoules d'IPA (lot 8IT11001, date de validité: 30/11/2021) fournies par le fabricant, Inosan Biopharma. L'IPA est composé de fragments d'immunoglobulines F(ab’)_2_ produits par immunisation de chevaux avec les venins *d'Echis ocellatus, E. pyramidum, E. leucogaster, Bitis gabonica, B. arietans, Naja haje, N. melanoleuca, N. nigricollis, N. pallida, Dendroaspis polylepis et D. jamesoni.* Le contenu de chaque ampoule neutralise au moins 250 doses létales pour une souris de 20 grammes du venin de chacune de ces espèces (= 250 DE_50_).

**Figure 1:**
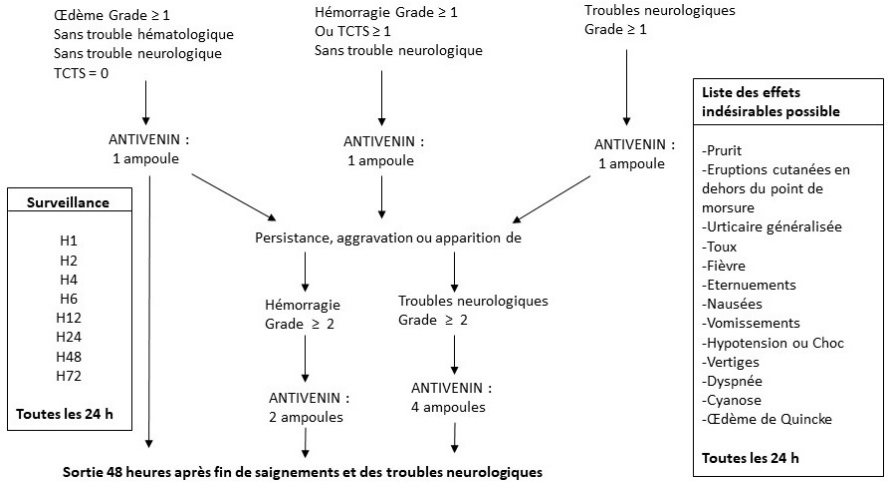
Algorithme de traitement utilisé au cours de cette étude Treatment algorithm used in the study

L'antivenin a été administré selon un protocole thérapeutique conçu dans le cadre de l’étude sur la base des recommandations de la SAV modifié avec l'accord de la direction médicale de l'hôpital (Fig. 1) après un avis favorable du Comité d’éthique de la Faculté des sciences de la santé de l'Université d'Abomey-Calavi. Tous les patients présentant une envenimation vipérine (douleur + œdème, avec ou sans anomalie du TCTS, avec ou sans saignement, ou troubles neurotoxiques), recevaient à leur arrivée une dose de 250 DE_50_ d'antivenin (1 ampoule d'IPA) en intraveineuse directe lente (IVDL). La première ampoule administrée était achetée par le patient, ou par l'hôpital si le patient ne pouvait pas la payer. Toutes les autres ampoules en cas de renouvellement, ainsi que les traitements adjuvants (transfusion sanguine notamment) étaient à la charge du projet.

Une nouvelle évaluation clinique et un TCTS étaient effectués 2 heures après la première administration (H_2_), puis 4 heures (H_4_) et 6 heures (H_6_) après la première administration d'IPA. À chaque évaluation (H_2_, H_4_, H_6_, H_12_, H_24_ puis toutes les 24 heures), l’évolution de l'envenimation était mesurée selon le même score clinique qu’à l'entrée. En cas de persistance, d'aggravation ou d'apparition des saignements, une nouvelle administration d'IPA, à la dose de 500 DE_50_ (2 ampoules d'IPA), était faite en IVDL. En cas de persistance, d'aggravation ou d'apparition de troubles neurotoxiques, une nouvelle administration d'IPA, à la dose de 1 000 DE_50_ (4 ampoules d'IPA), était faite en IVDL. L'administration de substituts sanguins (transfusion sanguine, plasma frais congelé, etc.) pouvait être associée à celle d'antivenin.

En cas de saignements tardifs ou de récidives de saignements au-delà de la 24^e^ heure, l'antivenin était administré à raison de 2 ampoules d'IPA par jour. Avant la 24^e^ heure, les antifibrinolytiques (acide tranexamique, acide ε-aminocaproïque) et les corticoïdes n’étaient pas utilisés pour ne pas perturber l’évaluation de l'efficacité de l'antivenin.

L’œdème, même en cas d'aggravation secondaire, n’était pas un critère de ré-administration de l'IPA après la première administration de H_0_. La persistance d'un TCTS anormal entre H_2_ et H_24_ n’était pas un critère de réadministration de l'IPA après la première administration de H_0_.

### Surveillance des évènements indésirables

Avant la première administration d'IPA, le patient faisait l'objet d'une recherche systématique selon un questionnaire standardisé de tous les symptômes compatibles avec un effet indésirable de l'antivenin [11]. Après chaque administration d'IPA, une surveillance clinique continue pendant 1 heure enregistrait les évènements indésirables.

Les effets indésirables tardifs, notamment les signes d'une maladie sérique, étaient recherchés lors d'une consultation à l'hôpital au cours de la 2^e^ semaine après le début du traitement ou par appel téléphonique.

### Critères de jugement principal

L'efficacité d'IPA était mesurée par l'absence de saignements et/ou la normalisation du TCTS au cours des 24 heures suivant l'administration initiale. En se référant aux études précédentes utilisant des antivenins composés de F(ab’)_2_, l'arrêt des saignements est attendu chez environ 50% des patients à H_2_, deux tiers à H_12_ et plus de 90% des patients à la 24^e^ heure. La normalisation du TCTS apparaît chez un quart des patients à H_2_, la moitié à H_12_ et plus de 80% à H_24_ [7, 8, 10, 12].

L'incidence attendue des effets indésirables imputables à l'IPA est inférieure à 10%.

### Critère de jugement secondaire

Le protocole allégé est attendu pour être aussi efficace que le protocole original de la SAV. L'efficacité de ce protocole sera comparée à celle du protocole de la SAV dans l’étude de Chippaux *et al.* réalisée à Tanguiéta en 2014 [3].

### Analyse statistique

Les variables qualitatives ont été exprimées sous forme de pourcentage et les variables quantitatives sous forme de moyenne, avec un intervalle de confiance (IC) à 95%, ou de médiane, avec les interquartiles (IIQ) à 25% et 75%, selon que la distribution de la variable était gaussienne ou non. Pour l'analyse de survie (Kaplan-Meier), les variables TCTS et Saignement ont été recodées de façon binaire avec comme modalité 0 pour TCTS normal et 1 pour TCTS anormal, et 0 pour absence de saignement et 1 pour présence de saignement. Les pourcentages de TCTS anormal et de présence de saignement ont été calculés pour chaque temps (H_0_, H_2_, H_4_, H_6_, H_12_, H_24_). Les incidences cumulées d'arrêt de saignement et de normalisation du TCTS ont été estimées à partir d'une courbe de Kaplan-Meier.

Les comparaisons ont été réalisées à l'aide d'un test de χ^2^. Il n'y a pas eu d'analyse multivariée ni de prise en compte de comparaisons multiples.

Les résultats ont été comparés avec 2 études antérieures, l'une où un antivenin similaire (Antivipmyn Africa) a été utilisé [10] et une seconde où le même antivenin (IPA) a été administré [7], tous les deux à la dose initiale de 2 ampoules.

L'analyse statistique a été conduite à l'aide des logiciels Excel 2016 et R version 4.1.2.

Le seuil de significativité est de p = 0,05.

### Dispositions éthiques

Les patients ont été inclus sur la base de leur consentement libre et éclairé. Les données recueillies ont été gardées confidentielles. Les autorisations administratives ont été obtenues auprès de la direction de l'hôpital de zone de Tanguiéta.

### Résultats

Notre étude s'est déroulée du 31 juillet au 31 octobre 2019. Nous avons reçu 53 cas de morsures de serpents. Dix patients n'ont pas été inclus pour les raisons suivantes: 3 ont reçu un antivenin avant admission, 6 ont consulté plus de 7 jours après la morsure, et 1 ne présentait aucun signe clinique ou biologique d'envenimation. Tous ces patients ont néanmoins été pris en charge en fonction de leur état clinique. La taille définitive de notre échantillon était de 43 patients.

### Aspects socio-démographiques (Tableau II)

L’âge médian était de 21 [IIQ: 18-31] ans. Le sexe masculin était le plus représenté avec un sex-ratio de 1,5. Les cultivateurs représentaient près de la moitié des patients (48%). Le pied était le siège de prédilection des morsures (84%).

**Tableau II:** Description des patients inclus et présentation clinique à l'admission Demography of patients included and clinical presentation on admission

Variables	N = 43
**Âge**	21 (18,29)1
sexe	
femmes	17 / 43 (40%)
hommes	26 / 43 (60%)
**Siège de morsure**	
membres inférieurs	38 / 43 (88%)
membres supérieurs	5 / 43 (12%)
**Délai d'admission**	1 (0,2)^1^
**Durée d'hospitalisation**	4 (2,6)^1^
**Œdème**	
non	5 / 43 (12%)
stade 1	13 / 43 (30%)
stade 2	19 / 43 (44%)
stade 3	6 / 43 (14%)
**Hémorragies^2^**	
0	25 / 43 (58%)
1	1 / 43 (2,3%)
2	16 / 43 (37%)
3	1 / 43 (2,3%)
**TCTS^2^**	
0	11 / 43 (26%)
1	8 / 43 (19%)
2	24 / 43 (56%)
**Nombre de ré-administrations**	
0	30 / 43 (70%)
1	9 / 43 (21%)
2	4 / 43 (9,3%)

1 Médiane (25%,75%); n / N (%)

2 Voir le détail du score des hémorragies dans le Tableau I

### Aspects cliniques et paracliniques

Le délai d'admission a varié de moins d’1 heure à 7 jours avec une médiane de 1 [IIQ: 0-2] jour (Tableau II).

Trente-huit patients présentaient un œdème (88%). Un syndrome hémorragique a été diagnostiqué (saignement actif extériorisé et/ou TCTS anormal) chez 32 patients (74%) à l'admission. Aucun cas de nécrose n'a été enregistré. Aucun patient n'a présenté de manifestations neurologiques.

La durée d'hospitalisation a été comprise entre 0 et 19 jours avec une médiane de 4 [IIQ: 2-6,5] jours.

### Aspects thérapeutiques

Vingt-cinq patients (58%) ont reçu un traitement traditionnel (notamment applications de plantes médicinales et scarifications multiples) avant leur admission.

Une ré-administration d'IPA a été nécessaire chez 13 (30%) patients (Tableau II). Le nombre d'ampoules administrées a été de 77 (moyenne = 1,8 ± 0,4; médiane = 1[IIQ: 1-3].

### Évaluation de l'efficacité (Tableau III)

#### Évolution de l'efficacité biologique à partir du TCTS

Trente-deux patients (74%) ont présenté un TCTS anormal. Il s'est normalisé en 24 [IIQ: 4-72] heures.

**Tableau III:** Évolution des indicateurs d'hémostase après l'administration d'IPA Evolution of hemostasis indicators after IPA administration

Critère	N total	H_0_	H_2_	H_12_	H_24_	H_48_
TCTS normal	43	11	17	30	33	37
TCTS anormal		32	26	13	10	6
Pas de saignement	43	25	30	42	41	41
Saignements cliniques		18	13	1	2	2

**Figure 2:**
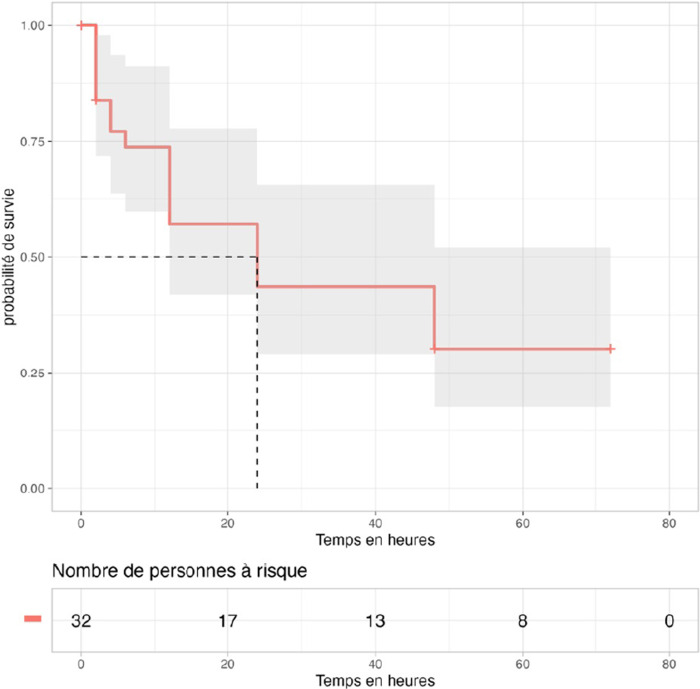
Évolution du TCTS après administration de l'antivenin. La zone grisée correspond à l'intervalle de confiance à 95% (N = 43) Evolution of WBCT after IPA administration. The gray area corresponds to the 95% confidence interval limits (N=43)

À H_2_, 26 (81%) patients avaient encore un TCTS anormal. À H_24_, 69% des patients avaient normalisé leur TCTS. La Figure 2 montre l’évolution des troubles de la coagulation après administration de l'antivenin, chez les patients ayant un trouble de coagulation à l'admission.

#### Évolution de l'efficacité clinique à partir des saignements

Vingt-et-un (49%) patients ont présenté un saignement à un moment quelconque de leur hospitalisation. Le délai médian d'arrêt des saignements était de 6 [IIQ: 4-12] heures.

Dix-huit (42%) patients saignaient à leur admission. Leur saignement s'est arrêté à H_2_ pour 11 d'entre eux, et chez tous à H_12_. Cependant, chez 3 des 11 patients dont les saignements s’étaient arrêtés à H_2_, une récidive du saignement est survenue, à H_4_ pour deux d'entre eux et H_24_ pour le troisième.

**Figure 3:**
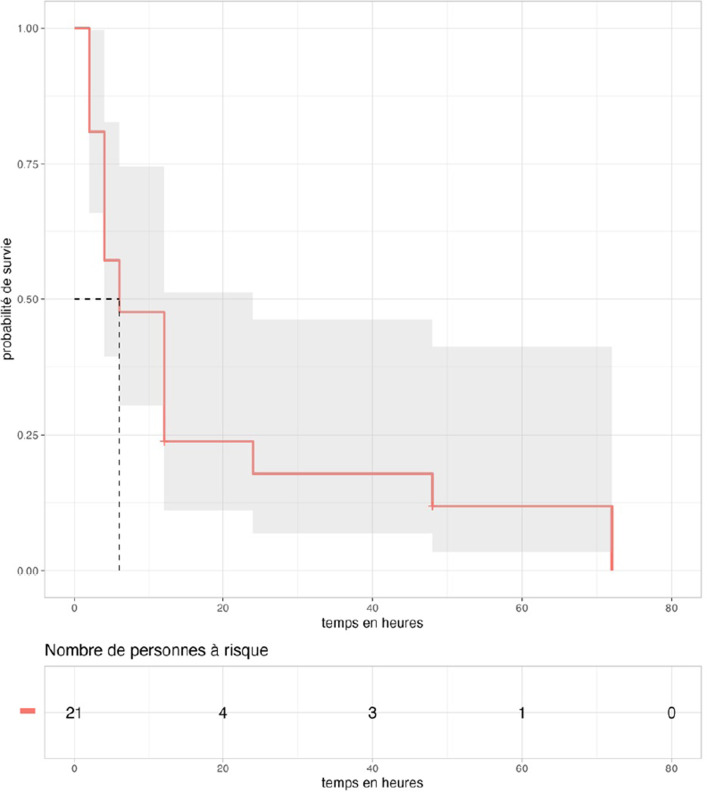
Évolution des saignements après administration de l'antivenin. La zone grisée correspond à l'intervalle de confiance à 95% (N = 43) Evolution of bleedings after IPA administration. The gray area corresponds to the 95% confidence interval limits (N=43)

En outre, 3 (7%) des patients qui ne saignaient pas à l'entrée, ont présenté des saignements tardifs après 24 heures malgré l'administration d'une ampoule d'IPA à l'admission. La Figure 3 montre l’évolution des saignements après administration de l'antivenin, chez les patients ayant présenté des saignements à un moment quelconque durant leur hospitalisation.

La fréquence des ré-administrations est précisée dans le Tableau II.

### Létalité et caractéristiques des patients décédés

Deux patients (5%) sont décédés des suites de l'envenimation.

Le premier patient est arrivé 10 heures après la morsure. Il avait été mordu au pied gauche. Il avait procédé à un traitement traditionnel (applications de plantes médicinales + multiples scarifications). Devant une aggravation de son état, il consulte dans un centre de santé qui le réfère à l'hôpital de zone Saint Jean de Dieu de Tanguiéta pour une meilleure prise en charge. À l'admission, il était dans le coma avec un score de Glasgow à 6/15. Il avait un pouls filant et des extrémités froides. Il avait un saignement local et aux points de scarifications. L'hémogramme retrouvait un taux d'hémoglobine à 6,5 g/dl, avec une hyperleucocytose à 37,6 g/l et un taux de plaquettes à 165 g/l. Le TCTS était incoagulable. Il a bénéficié d'une réanimation faite d'oxygénothérapie et de remplissage vasculaire. Il n'a pas été possible de réaliser une transfusion faute de sang disponible. Il est décédé 10 minutes après l'administration de l'IPA.

Le second patient a été admis 7 jours après la morsure. Il avait été mordu au pied gauche. Il avait aussi recouru à un traitement traditionnel (plante médicinale + scarifications) sans succès. À l'admission, il était très agité avec des propos incohérents, et présentait un saignement aux points de pénétration des crochets. La tension artérielle était de 120/60 mm Hg à l'entrée. Le TCTS était incoagulable et l'hémogramme réalisé retrouvait un taux d'hémoglobine à 6,6 g/dl, une hyperleucocytose à 19 g/l et un taux de plaquettes à 454 g/l. L’évolution a été marquée par une altération progressive de l’état de conscience, puis le décès 4 h après l'administration de l'IPA alors qu'il était en attente d'une transfusion sanguine.

### Intolérance à l'antivenin

Les différentes réactions immédiates développées et compatibles avec une allergie à l'antivenin, allaient de la nausée à l’état de choc. Sept patients (16%) ont présenté des signes d'intolérance (Tableau IV). Nous avons recontacté 30 patients (70%) 3 semaines à 2 mois après le traitement. Aucun d'entre eux n'a signalé une manifestation clinique pouvant faire évoquer une intolérance tardive, y compris une maladie sérique.

**Tableau IV:** Description des évènements indésirables observés après l'administration d'IPA Description of adverse events observed after IPA administration

Délai morsure IPA* (heures)	Délai IPA* EI (minutes)	Symptômes EI**	Traitement EI**	Renouvellement IPA*	Gravité	Imputabilité à IPA*
10	5	Prurit, éternuements, hypotension, dyspnée, coma	Dexaméthasone, oxygène	Oui (avec manifestations modérées)	Grave	Possible
48	3	Prurit, toux, vertiges	Dexaméthasone	Oui (avec manifestations modérées)	Bénin	Probable
10	2	Prurit, nausées, éternuements,	Dexaméthasone	Oui (avec manifestations modérées)	Bénin	Probable
48	4	Nausées	Aucun	Non	Bénin	Probable
4	3	Nausées	Aucun	Non	Bénin	Probable
2	3	Toux, éternuements	Dexaméthasone	Non	Bénin	Probable
24	3	Fièvre, prurit, toux, dyspnée	Aucun	Non	Bénin	Probable

* IPA = administration d'Inoserp^®^ Pan-Africa

** EI = évènement indésirable

## Discussion

Nos résultats confirment la forte prévalence des syndromes hémorragiques dans la région de Tanguiéta, comme cela avait été rapporté lors des études béninoises précédentes [7, 10]: sur 43 patients inclus, 47% présentaient des saignements actifs et 74% un TCTS anormal traduisant un trouble de l'hémostase (Tableau V).

**Tableau V:** Comparaison de la présentation du syndrome hémorragique au cours des études cliniques réalisées à Tanguiéta Comparison of the presentation of haemorrhagic syndrome in clinical studies carried out in Tanguiéta

Variables	Nord Bénin 2006 [10]	Nord Bénin 2013 [7]	Tanguiéta 2019 (cette étude)
Nombre de patients	289	100	43
Délai d'admission (heures)	24*	24 [9-48]**	24 [0-48]**
Âge moyen (ans)	22	25	24
Sex-ratio (H/F)	2,4	3,8	1,5
% saignements	48	56	47
% TCTS anormaux	75	90	74
Dose moyenne (ampoules)	1,9	1,7	1,8
Durée hospitalisation (jours)	3	5,3	4,8
Létalité (%)	3,1	4	4,7

* Moyenne

** Médiane [IIQ]

Dans notre série, nous avons 15% de saignements retardés et 15% de récidives des saignements après rémission. Lors de l’étude clinique réalisée dans plusieurs hôpitaux du nord du Bénin avec un antivenin différent de l'IPA, il avait été rapporté la survenue d'un saignement retardé chez 20% des patients et d'une récidive des saignements après une rémission chez 29% [10]. En revanche, sur 60 patients traités au cours de l’étude clinique de l'IPA, il n'a pas été observé de saignement retardé ni de récidive [7]. Larréché *et al.* (2018) rapportaient aussi des cas de récidives de saignements et de saignements tardifs dans leur étude menée à Djibouti après morsure par *Echis pyramidum,* une espèce voisine d'E. *ocellatus* qui se trouve au nord du Bénin [13].

Trois hypothèses, d'ailleurs non exclusives, peuvent être formulées:

- En premier lieu, il pourrait s'agir d'une dose d'antivenin insuffisante. Une fois inoculé, le venin diffuse par voie lymphaticosanguine et s’équilibre entre les différents compartiments (sang et organes profonds). L'administration intraveineuse d'antivenin élimine le venin du compartiment plasmatique, ce qui modifie l’équilibre de concentration du venin et crée un flux à partir des compartiments profonds vers le sang [17]. Administré en grande quantité, l'antivenin élimine rapidement la totalité du venin présent dans l'organisme. En revanche, une dose trop faible réduit la concentration de venin sans l’éliminer totalement, ce qui pourrait expliquer la poursuite des troubles de l'hémostase, surtout si une quantité élevée de venin a été injectée par le serpent. La mesure de la veninémie nous permettrait de confirmer cette hypothèse. Dans ce cas, il faudrait réajuster la dose d'antivenin.
- Deuxièmement, le caillot pourrait ne pas se former en raison d'un défaut de facteurs de coagulation. Le venin agit sur les plaquettes sanguines et la coagulation plasmatique consommant, notamment, le fibrinogène [13, 14]. La restauration des facteurs de coagulation demande plusieurs jours après l’élimination totale du venin. Un dosage des différents facteurs de coagulation, principalement les plaquettes et le fibrinogène, permettrait de répondre à cette question. Si cette hypothèse était vérifiée, la transfusion de sang total, de fractions sanguines ou de plasma frais congelé pourrait constituer une solution acceptable en raison de son coût raisonnable, sous réserve que la production contrôle effectivement tout risque d'infection.
- La 3^e^ hypothèse est celle d'une hyperfibrinolyse secondaire survenant tardivement et persistant plusieurs jours malgré l'administration d'antivenin [13, 16]. L'hyperfibrinolyse tardive serait une réponse de l'organisme à l'action hypercoagulante du venin et, peut-être, à la dégradation de l'endothélium dont est responsable le venin de Viperidae [18]. La destruction du caillot sanguin au fur et à mesure de sa formation après l'administration d'antivenin, expliquerait le prolongement des troubles de la coagulation marqués par un TCTS anormal, des saignements retardés et des récidives de saignements qui ne seraient donc pas liés à une inefficacité de l'antivenin. L'administration précoce d'un antifibrinolytique à dose élevée serait une réponse peu coûteuse qui, cependant, doit faire l'objet d'un essai clinique.

La létalité dans notre étude était de 5%, ce qui correspond à celles rapportées par les 2 autres études béninoises dont les patients avaient reçu 2 ampoules d'antivenin comme dose initiale, respectivement 3,1% et 4% [7, 10]. Il faut souligner que les deux patients décédés sont arrivés à l'hôpital plus de 10 heures après la morsure et présentaient une anémie décompensée et un état de choc. La létalité n'est pas un critère pertinent d'appréciation de l'efficacité, car il s'agit souvent de patients présentant des complications irréversibles apparues avant la prise en charge et sur lesquelles l'antivenin n'a que peu d'effets [7]. Le venin *d'Echis* induit une coagulopathie de consommation contre laquelle l'antivenin s'est montré efficace [2, 14]. Elle pourrait, cependant, être remise en cause, au moins chez certains patients [2], notamment, par une dose insuffisante d'antivenin.

Cela justifie l'administration d'antivenin dans les plus brefs délais, avant que ne se déclare la coagulopathie de consommation.

Le protocole allégé que nous avons utilisé, correspond à la pratique courante réalisée à l'hôpital Saint Jean de Dieu et dans de nombreuses autres formations sanitaires de la région. Il représente une économie significative du nombre d'ampoules utilisées, soit 77 ampoules (moyenne = 1,8 ± 0,4) au lieu des 101 ampoules (moyenne = 2,4 ± 0,2) qui auraient été nécessaires chez les mêmes patients traités selon le protocole standard avec une estimation conservatrice (p = 2,6·10^-4^). La différence représente une trentaine d'euros par patient, ce qui correspond à la moitié du revenu mensuel brut par Béninois selon la Banque mondiale. Il est plus facilement accepté par les familles et ménage le stock d'antivenins du centre de santé.

Toutefois, en comparaison avec les résultats obtenus lors de la précédente étude clinique réalisée à Tanguiéta [7], l'arrêt des saignements est retardé avec une fréquence significativement plus basse de l'arrêt des saignements pour le protocole allégé (67% à H_3_ dans la précédente étude *versus* 28% à H_2_ dans celle-ci; p = 0,008). Chippaux *et al.* (2015) avaient constaté un prolongement hautement significatif de la persistance des saignements chez les patients dont la dose d'antivenin avait été inférieure à celle prévue par le protocole en raison d’écarts au protocole [7]. La normalisation du TCTS est également différée significativement à H_2_/H_3_, H_12_ et H_24_, respectivement 47% à H_3_ dans l’étude précédente *versus* 19% à H_2_ dans celle-ci (p = 0,008), 92% *versus* 59% à H_12_ (p = 2·10^-4^) et 98% *versus* 69% à H_24_ (p = 1·10^-5^). En outre, le nombre de ré-administrations d'antivenin après H_2_ est supérieur dans le protocole allégé à celui du protocole standard, respectivement 13 sur 43 (30%) patients traités *versus* 4 sur 59 (7%) patients (p = 0,006).

Il n'y a pas de différence significative entre l'incidence de signes d'intolérance précoce à l'antivenin dans cette étude (16%) par rapport à la précédente (11%; p = 0,79). Cependant, l'imputabilité reste difficile à apprécier, car l'antivenin était administré en même temps (H_0_) que le sérum antitétanique qui peut aussi entraîner des effets indésirables.

## Conclusion

Le protocole que nous avons utilisé dans cette étude se révèle relativement efficace et bien toléré. Cependant, malgré une économie significative du nombre d'ampoules dans le protocole allégé (dont le coût serait réduit d'environ 25%), il apparaît une insuffisance d'efficacité. Le délai de normalisation de la coagulation sanguine est significativement plus long qu'avec le protocole standard. Ce délai peut être délétère et faire courir un risque de complications chez près d'un tiers des patients, ce qui ne nous semble pas raisonnable au regard de l’économie potentielle. Le financement du traitement s'avère indispensable pour assurer une prise en charge efficiente des envenimations en Afrique subsaharienne [9].

## Remerciements

Nous remercions Inosan Biopharma pour la mise à disposition des doses d'Inoserp’ Pan-Africa nous ayant permis de réaliser cette étude. Notre gratitude va à l'ensemble du personnel de l'hôpital Saint Jean de Dieu qui nous a aidés tout au long de notre travail et aux patients ainsi qu’à leur famille pour leur participation.

## Financement

Cette étude a été financée par l'Institut de recherche pour le développement et l'Institut de recherche clinique du Bénin.

## Contribution des auteurs

Jean-Philippe CHIPPAUX et Achille MASSOUGBODJI ont élaboré le protocole, supervisé l’étude clinique et les analyses, et validé la version finale de l'article.

Harold TANKPINOU a réalisé l’étude de terrain, analysé les données et rédigé la première version de cet article.

Pierre FACHEHOUN et Giambattista PRIULI, médecins à l'hôpital Saint Jean de Dieu, ont suivi les patients au cours de leur hospitalisation et assuré leur prise en charge médico-chirurgicale.

## Liens d'intérêts

Les auteurs ne déclarent aucun conflit d'intérêts.
